# Short and long-term impact of four sets of actions on acute ischemic stroke management in Rhône County, a population based before-and-after prospective study

**DOI:** 10.1186/s12913-020-05982-0

**Published:** 2021-01-04

**Authors:** A. M. Schott, A. Termoz, M. Viprey, K. Tazarourte, C. Della Vecchia, E. Bravant, N. Perreton, N. Nighoghossian, S. Cakmak, S. Meyran, B. Ducreux, C. Pidoux, T. Bony, M. Douplat, V. Potinet, A. Sigal, Y. Xue, L. Derex, J. Haesebaert

**Affiliations:** 1 Université de Lyon, Université Claude Bernard Lyon 1 - HESPER EA 7425, 8 Avenue Rockefeller, 69008 Lyon, France; 2 Hospices Civils de Lyon, Pôle de Sante Publique, Lyon, France; 3 Emergency Department – HEH, Hospices Civils de Lyon, Lyon, France; 4 Hospices Civils de Lyon, Comprehensive Stroke Center, Hôpital Pierre Wertheimer, Bron, France; 5 Hôpital Nord Ouest, Primary Stroke Center, Villefranche-sur-Saône, France; 6 Emergency Department, Hôpital St Joseph St Luc, Lyon, France; 7 Emergency Department, Hôpital Nord Ouest, Villefranche-sur-Saône, France; 8 Emergency Department, Hospices Civils de Lyon, Hôpital Lyon Sud, Pierre Bénite, France; 9 Emergency Department, Hospices Civils de Lyon, Hôpital Croix Rousse, Lyon, France

**Keywords:** Ischemic stroke, Organization, Reperfusion therapy, Cohort study, Time-to-treatment, Emergency medical services, Health services research

## Abstract

**Background:**

Optimizing access to recanalization therapies in acute ischemic stroke patients is crucial. Our aim was to measure the short and long term effectiveness, at the acute phase and 1 year after stroke, of four sets of actions implemented in the Rhône County.

**Methods:**

The four multilevel actions were 1) increase in stroke units bed capacity and development of endovascular therapy; 2) improvement in knowledge and skills of healthcare providers involved in acute stroke management using a bottom-up approach; 3) development and implementation of new organizations (transportation routes, pre-notification, coordination by the emergency call center physician dispatcher); and 4) launch of regional public awareness campaigns in addition to national campaigns. A before-and-after study was conducted with two identical population-based cohort studies in 2006–7 and 2015–16 in all adult ischemic stroke patients admitted to any emergency department or stroke unit of the Rhône County. The primary outcome criterion was in-hospital management times, and the main secondary outcome criteria were access to reperfusion therapy (either intravenous thrombolysis or endovascular treatment) and pre-hospital management times in the short term, and 12-month prognosis measured by the modified Rankin Scale (mRS) in the long term.

**Results:**

Between 2015–16 and 2006–7 periods ischemic stroke patients increased from 696 to 717, access to reperfusion therapy increased from 9 to 23% (*p* < 0.0001), calls to emergency call-center from 40 to 68% (*p* < 0.0001), first admission in stroke unit from 8 to 30% (*p* < 0.0001), and MRI within 24 h from 18 to 42% (*p* < 0.0001). Onset-to-reperfusion time significantly decreased from 3h16mn [2 h54-4 h05] to 2h35mn [2 h05-3 h19] (*p* < 0.0001), mainly related to a decrease in delay from admission to imaging. A significant decrease of disability was observed, as patients with mild disability (mRS [0–2]) at 12 months increased from 48 to 61% (*p* < 0.0001). Pre-hospital times, however, did not change significantly.

**Conclusions:**

We observed significant improvement in access to reperfusion therapy, mainly through a strong decrease of in-hospital management times, and in 12-month disability after the implementation of four sets of actions between 2006 and 2016 in the Rhône County. Reducing pre-hospital times remains a challenge.

## Background

The benefit of recanalization therapies in patients with ischemic stroke is strongly time-dependent, earlier intervention achieving better outcomes [1–3]. Stroke systems of care need to minimize latency to assessment and initiation of treatment, before brain injury becomes irreversible.

For acute ischemic stroke patient management, timely restoration of blood flow through intravenous thrombolysis (IVT) or endovascular treatment (EVT) is the first priority. Several actions were defined by the European Academy of Neurology and European Stroke Organization to improve effectiveness of acute stroke care organization throughout the overall process of pre-hospital care [4]. Those actions target each step of the patient’s journey and include: public awareness programs to increase the number of patients calling the centralized emergency call center (ECC) in a timely manner, using a unique telephone number (#15 in France or #112 in Europe); training of emergency staff to accurately identify acute stroke patients using validated tools; establishment of clear transportation routes to the nearest suitable hospital; organizational procedures such as pre-notification or direct arrival in the radiology department; and implementation of registries to monitor key performance indicators. These actions complement the development of stroke units and stroke centers since hospitalization in such centers reduces disability, institutional care, and death by 20% [5]. At the national level a survey estimated in 2009 that only 1% of patients underwent thrombolysis [6]. Stroke prevention and management were therefore identified as a national priority for the years 2010–2014, with the aim to implement actions in each health territory to reduce ischemic stroke-related mortality and morbidity [7]. Stroke units and more recently stroke centers were developed to reduce geographical inequities and improve access to specialized care and reperfusion therapy. At a regional level, in 2006–7 (Nov 2006-June 2007), the population-based cohort study “AVC69” on all consecutive patients with a suspicion of stroke admitted to any emergency department or stroke unit of the Rhône County showed that only 9% of all ischemic stroke patients had access to thrombolysis [8]. Four sets of actions were developed in the county, and in 2015–16 (Nov 2015-June 2016), a second cohort study (STROKE69) with an identical design as the one conducted in 2006–7 (AVC69) was performed. The aim of the present study was to assess the effectiveness of all actions implemented on short and long-term outcomes, at the acute phase and 1 year after the stroke. The primary outcome criterion was admission-to-imaging time, and the main secondary outcome criteria was access to reperfusion therapy, either IVT or EVT in the short term and 12-month prognosis measured by the modified Rankin Scale (mRS) in the long term.

## Methods

We used the Strengthening the Reporting of Observational Studies in Epidemiology (STROBE) Statement [9] to guide the reporting.

### Design and population

The present study is a before-and-after study with two identically designed cohort studies. AVC69 study was a population-based prospective study conducted between November 2006 and June 2007. All patients aged over 18 years with a suspected acute stroke, admitted to any emergency department or stroke unit or stroke center of the Rhône County were identified by emergency physicians in emergency departments and neurologists in stroke units and included in the cohort. Definitive diagnosis was based on cerebral imaging (computed tomography scan or magnetic resonance imaging) and was confirmed by a neurologist (L Derex) based on imaging and examination of the patients or their hospital record. Patients were followed-up through a phone call 12 months after their stroke. STROKE69 study was a similarly designed population-based prospective cohort study conducted between November 2015 and June 2016 in the Rhône County.

### Sets of actions implemented between the two periods of observation

**Fig. 1:**
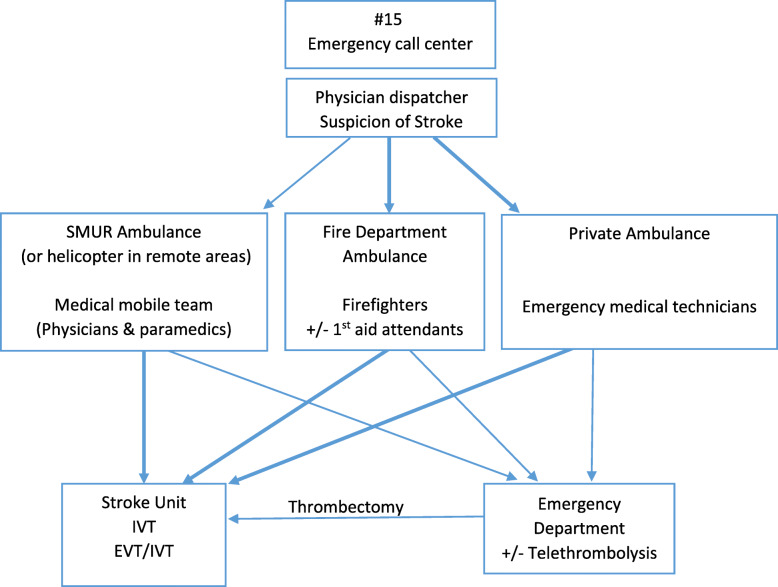
Organization of acute stroke emergency care in France. legend: Thicker arrows represent the best theoretical pathways for most effective management as recommended by French guidelines. Abbreviations: SMUR, *Service Mobile d’Urgence et de Réanimation* (system of highly medicalized transportation)

Organization of acute stroke emergency care in France is described in Fig. 1. The medical dispatcher of the emergency call center (ECC) “15”, decides one of three distinct types of transport depending on clinical severity, estimation of transportation time and availability, either private ambulance, firefighters, or SMUR (service mobile d’urgence et de reanimation ie ‘Mobile Hospital Unit’), a system of highly medicalized transportation, if the situation is not stable and requires the support of emergency physicians on site. Private medical transport companies (ie ambulances) may participate to emergency transportation if they have an agreement with the national health insurance fund and only on physician prescription. For most cases of ischemic stroke, the patients clinical condition does not require a medical team on site but rather an urgent transportation to the nearest stroke unit or stroke center, thus firefighters transportation, which is usually faster than both SMUR and ambulances, is particularly recommended. In the Rhône County we found in a previous work that fire brigade access capability was greater than SMUR because of a larger number and a better geographical repartition of fire stations in the territory [10]. In 2006–7 there were seven emergency departments the Rhone County, and one stroke unit (in Lyon). Following AVC69 study, four sets of actions were undertaken to improve the organization of “Acute Stroke Pathway” (sets of actions #2 and #4, which are complex interventions, are also described with the Template for Intervention Description and Replication (TIDieR) Checklist in Appendix 1).

#### First set of actions: increasing stroke units’ capacity

The first set of actions concerned the increase in stroke units’ capacities. The Lyon stroke unit capacity increased from 6 to 12 beds and became a stroke center with the development of EVT activity, and a stroke unit of 4 beds was created in Villefranche-sur-Saône.

#### Second set of actions: improving professionals’ knowledge and skills

The second set of actions aimed at improving knowledge and skills of the different categories of healthcare professionals involved in acute stroke care using a bottom-up approach as heterogeneous knowledge and skills among these healthcare professionals have been often observed, which may cause longer delays [11]. We also observed within a randomized trial that beyond the technical skills needed to recognize stroke as such and as an emergency, awareness of the impact of any additional delay on patients’ final disability was low particularly in emergency healthcare professionals [12]. Within this randomized trial, an intervention was developed based on an interdisciplinary training using specifically designed videos, simulated patients, and feed-back to increase awareness regarding the benefits of a rapid management during acute phase on patients’ long-term disability (mms://vod.univ-lyon1.fr/AVC/avc.asf). A bottom-up interdisciplinary approach was also set-up to identify effective local pathways and procedures. This intervention was delivered during a one-day training session by neurologists and emergency physicians to some referent emergency physicians, emergency nurses and radiologists of the participating centers who in turn had to implement the intervention in their department. Additionally to this intervention conducted within a randomized trial in 2010–2011, feed-back and morbidity-mortality reviews were implemented during emergency department staff rounds as well as an immersive program for emergency department physicians in the stroke unit of Lyon, which are still ongoing. This set of actions aimed at improving knowledge, awareness, technical skills and self-confidence of healthcare professionals involved in the acute stroke care pathway.

#### Third set of actions: re-organizing acute stroke care pathway

The third set of actions consisted in overall re-organization of the care pathway. Based on a specific research program we established clear transportation routes to the nearest suitable hospital [10], as well as pre-notification of the in-hospital stroke team, direct access to the stroke unit or the radiology department, and overall coordination by only one person, the ECC physician dispatcher. These actions were conducted between 2010 and 2016.

#### Fourth set of actions: regional public information campaign

The fourth set of actions was the development of the ReACT information campaign that aimed to improve stroke recognition in the general population and prompt call to ECC. The content and the form of the messages were based on the results of a previous qualitative study and were designed by a multidisciplinary team involving stroke unit neurologists, ECC physicians, public health researchers, pharmacists, psychologists, communication professionals, and representatives of patients’ associations, using a user-centered approach [13]. The campaign targeted three aspects, recognition of stroke symptoms, urgency of the situation and the need to call ECC. Stroke symptom recognition was based on the 3 FAST-symptoms (Face dropping, Arm weakness, Speech disturbance) and the urgency of the situation and the need to call EMS were illustrated by posters and video with actors simulating different situation of strokes. The diffusion strategy consisted in one public event on World Stroke Day in Lyon city-center, followed by a 2-month multi-media campaign. The event proposed a press conference and an information booth animated by different health professionals involved in stroke management and stroke patients. This campaign was launched in 2014–2015 in addition to the annual national public campaign and its impact was evaluated within a before-and-after study with a control group [14].

### Data collection

Every week research assistants identified suspected stroke cases through the emergency department registries and the stroke unit/stroke centers files to prevent any selection bias. They collected data on patients’ characteristics, outcome measures and all variables related with the acute phase management within hospital files. They called by phone stroke survivors 12 months after their stroke, to evaluate the modified Rankin score (mRS). Definitive diagnosis was based on cerebral imaging (computed tomography scan or magnetic resonance imaging) and was confirmed by a neurologist specialized in stroke management (L Derex).

The National Institute of Health Stroke Scale (NIHSS) score was too rarely performed by the emergency department physicians in the AVC69 cohort 2006–7 to be accurately analyzed and thus we present only the NIHSS observed in STROKE69 cohort. Main outcome measures were pre- and in-hospital management times and the proportion of acute ischemic stroke patients receiving a reperfusion technique (IVT and/or EVT). Other secondary outcomes were the number of patients calling ECC, first admission in a stroke unit, MRI performed within 24 h after admission, hospitalization in a stroke unit during the care pathway, and 12-month prognosis measured by the modified Rankin Scale (mRS).

### Statistical analyses

Sample size calculation for STROKE69 was based on the hypothesis of a 15% reduction in admission-to-imaging time between the two periods, corresponding to a 30-min decrease of the admission-to-imaging time observed in AVC69 cohort, which was 3h29mn. Applying a 5% alpha risk and an 85% power, the inclusion of 1300 confirmed cases of stroke was necessary. Quantitative variables were described using median and interquartile range (IQR). The NIHSS score was categorized as recommended into 4 categories: < 5 (minor stroke), 5–14 (moderate stroke), 15–20 (moderate to severe stroke), and > 20 (severe stroke). The modified Rankin was dichotomized into patients with no disability or slight disability but still able to look own affairs without assistance (mRS 0–2) versus patients with moderate disability requiring some help or more severe disability [15]. Categorical variables were described using frequencies and percentages. There was no replacement of missing data. Outcome variables were compared between the 2006–7 and 2015–16 cohorts using Chi-squared test for categorical characteristics and Student t-test for quantitative characteristics. Logistic regression models with access to reperfusion therapy (IVT/EVT) as the outcome variable and year of the study as explanatory variable were adjusted on age and sex. They were also adjusted on ECC call to be able to distinguish the effect of a possible increased awareness of the public from an improvement of healthcare organization. Statistical analyses were conducted with a 5% threshold for statistical significance for bilateral tests using the SAS software (version 9.3, SAS Institute Inc., Cary, NC, USA).

## Results

### Comparison of patient characteristics and care pathways

**Fig. 2:**
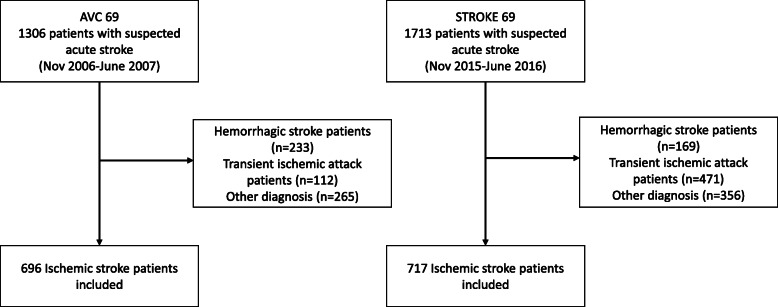
Study flow chart

Over a similar timeframe and a similar catchment area, 717 ischemic stroke patients were identified in 2015–16 compared to 696 in 2006–7, representing an increase of 3% (Fig. 2). Between 2006–7 and 2015–16 there was a slight decrease in hemorrhagic strokes, and an increase in transient ischemic attacks. Between 2006–7 and 2015–16, there was no significant difference in neither gender nor age of ischemic stroke patients in the Rhône County (Table 1). Regarding care pathway characteristics, more patients were transported by fire department ambulances in 2015–16 (58% versus 31%), and less by private ambulances (24% vs. 39%) or SMUR (Service Mobile d’Urgence et de Réanimation: system of highly medicalized transportation, 4% vs. 12%; Table 2).

**Table 1:** Ischemic stroke patients’ characteristics in the AVC69 and STROKE69 cohorts

Patients’ characteristics	AVC69 2006–7 ***n*** = 696	STROKE 69 2015–16 ***n*** = 717	p
Women, n (%) Missing data	362 (52) 0	360 (50) 0	0.50
Age, median [IQR] Missing data	79 [68–85] 0	79 [66–86] 0	0.99
NIHSS score^a^, n (%)			
< 5	–	341 (48)	
5–14	–	237 (33)	
15–20	–	76 (11)	
> 20	–	53 (8)	
Missing data	–	10 (1)	

*IQR* Interquartile Range, *NIHSS* National Institute of Health Stroke Scale

^a^NIHSS score was not available in the 2006–7 cohort

**Table 2:** Ischemic stroke patients management modalities

Stroke Management characteristics	AVC69 2006–7 ***n*** = 696	STROKE69 2015–16 ***n*** = 717	p
Place of Stroke, n (%)			
Home	483 (81)	505 (86)	
Family physician	71 (12)	20 (3)	
Highway	36 (6)	35 (6)	< 0.0001
Healthcare center	6 (1)	22 (4)	
Other	0	2 (0.3)	
Missing data	100 (14)	133 (19)	
Transportation type, n (%)			
Ambulance	224 (39)	151 (24)	
Firefighters	178 (31)	373 (58)	
*SMUR*	66 (12)	27 (4)	< 0.0001
Personal	104 (18)	85 (13)	
Other	1 (0.2)	2 (0.3)	
Missing data	123 (18)	79 (11)	
ECC call, n (%)	215 (40)	488 (68)	< 0.0001
Missing data	159 (23)	0	
Place of 1st admission, n (%)			
emergency department	615 (88)	502 (70)	
stroke unit	57 (8)	214 (30)	< 0.0001
ICU	12 (2)	1 (0.1)	
Other departments of neurology	12 (2)	0 (0)	
Missing data	0	0	
Hospitalization in stroke unit, n (%)	118 (17)	447 (62)	< 0.0001
Missing data	0	0	
Imaging within 24 h, n (%)			
CT alone	460 (77)	405 (57)	
MRI alone	52 (9)	221 (31)	< 0.0001
CT and MRI	51 (9)	79 (11)	
None	34 (6)	3 (0.4)	
Missing data	99 (14)	9 (1)	
Treatment, n (%)			
IVT alone	60 (9)	112 (16)	< 0.0001
EVT	0	55 (8)	
Any reperfusion therapy	60 (9)	167 (23)	< 0.0001
Missing data	0	0	
12-month prognosis, n (%)			
mRS [0–2]	251 (48)	319 (61)	< 0.0001
mRS [3–6]	276 (52)	202 (39)	
Missing data	169 (24)	196 (27)	

*CT* Computed Tomography, *ECC* Emergency Call Center, *EVT* Endovascular Treatment, *ICU* Intensive Care Unit, *IVT* Intravenous Thrombolysis, *MRI* Magnetic Resonance Imaging, *mRS* modified Rankin Scale; *SMUR*, *Service Mobile d’Urgence et de Réanimation* (system of highly medicalized transportation)

### Effectiveness indicators

Between the two periods, major changes occurred (Table 2). Regarding the acute phase, access to IVT or any reperfusion therapy (IVT and/or EVT) significantly increased, from 9 to 16% and from 9 to 23% respectively. Regarding secondary outcomes, significant increases were found in the number of patients calling ECC (40 to 68%), first admissions in a stroke unit (8 to 30%), MRI performed within 24 h (18 to 42%), and patients hospitalized in a stroke unit during the care pathway (17 to 62%). In the subgroup of patients admitted less than 4 h after symptom onset, 15% in 2006–7 vs. 27% in 2016–17 had access to thrombolysis (*p* < 0.0001), 15% vs. 39% had access to any reperfusion therapy (*p* < 0.0001), and 25% vs. 74% were hospitalized in a stroke unit (*p* < 0.0001). Regarding long-term effectiveness, patients with a mRS [0–2] at 12 month increased from 48 to 61%.

### Time indicators

Median time from symptom onset to hospital admission did not change significantly between 2006-7 and 2015–16 (*p* = 0.61), while delays between hospital admission and first imaging significantly decreased between the two periods (Table 3). This decrease was particularly visible on the shorter delay times, since for 25% of patients this delay was under 1 h12 in 2007 vs. 25 min in 2016. Times from imaging and from hospital admission to reperfusion therapy also significantly decreased, as well as total management times from symptom onset to reperfusion.

**Table 3:** Description of pre- and in-hospital management times of ischemic stroke patients

Stroke Management Times	AVC69 2006–7 ***n*** = 696	STROKE69 2015–16 ***n*** = 717	p
**Management times, median [IQR]**			
Symptom onset to ECC call	30mn [5mn-1 h15]	41mn [13mn-2 h46]	0.07
Missing data, n (%)	178 (83)	195 (40)	
ECC call to admission	1 h07 [54mn-1 h33]	1 h04 [50mn-1 h19]	0.10
Missing data, n (%)	169 (79)	110 (23)	
Symptom onset to admission	2 h21 [1 h25-4 h32]	2 h08 [1 h22-4 h48]	0.61
Missing data, n (%)	308 (44)	212 (30)	
Symptom onset to first imaging	4 h46 [3 h10-8 h45]	3 h46 [2 h03-8 h16]	< 0.0001
Missing data, n (%)	229 (41)	205 (29)	
Symptom onset to any reperfusion therapy	3 h16 [2 h54-4 h05]	2 h35 [2 h05-3 h19]	< 0.0001
Missing data, n (%)	24 (40)	25 (15)	
Symptom onset to IVT alone	3 h16 [2 h54-4 h05]	2 h40 [2 h05-3 h15]	< 0.0001
Missing data, n (%)	24 (40)	17 (15)	
Admission to first imaging	2 h10 [1 h12-3 h56]	1 h41 [25mn-3 h42]	< 0.0001
Missing data, n (%)	4 (1)	42 (6)	
Admission to any reperfusion therapy	1 h50 [55mn-2 h46]	50mn [29mn-1 h18]	0.0002
Missing data, n (%)	23 (38)	20 (12)	
Imaging to any reperfusion therapy	1 h10 [1 h-1 h38]	33mn [21mn-48mn]	< 0.0001
Missing data, n (%)	24 (40)	18 (11)	
Imaging to IVT alone	1 h10 [1 h-1 h38]	31mn [19mn-44mn]	< 0.0001
Missing data, n (%)	24 (40)	18 (16)	
**Patients with imaging < 60mn, N (%)**	103 (18)	251 (38)	< 0.0001
Missing data, n (%)	4 (1)	42 (36)	

*IQR* Interquartile Range, *ECC* Emergency Call Center, *IVT* Intravenous Thrombolysis

### Factors associated with access to reperfusion

As shown in Table 4, in the second period patients were 3.22 times more likely to receive a reperfusion therapy than in the first period. After adjustment for age, sex and calling ECC, ischemic stroke patients of the 2015–6 cohort were still more than twice more likely to receive a reperfusion therapy than patients in the 2006–7 cohort (adjOR = 2.23, 95%CI [1.55–3.20]). Male gender, which was associated with a higher chance of reperfusion therapy in the unadjusted model, was not significantly associated with this outcome once adjusted for age (adjOR = 1.08, 95%CI [0.78–1.50]). Ageing was associated with a decreased likelihood of accessing a reperfusion therapy (adjOR = 0.98, 95%CI [0.97–0.99]) and calling the ECC dramatically increased the probability of accessing reperfusion therapy (adjOR = 6.06, 95%CI [3.94–9.33]). This shows that the improvement in reperfusion rate was mostly associated with improvement in healthcare efficiency but also partly with more patients calling ECC.

**Table 4:** Logistic regression modeling the effect of the study period on the likelihood of reperfusion therapy

	Crude OR	95%CI	Adj OR	95%CI
STROKE 69 (vs AVC69)	3.22	2.35–4.42	2.23	1.55–3.20
Male gender (vs female)	1.34	1.01–1.79	1.08	0.78–1.50
Age	0.98	0.97–0.99	0.98	0.97–0.99
Calling ECC	7.09	4.65–10.80	6.06	3.94–9.33

## Discussion

Between 2006–7 and 2015–16, the proportion of ischemic stroke patients calling ECC and having access to reperfusion increased significantly, and in-hospital management times were significantly reduced. Altogether, these results suggest an effective improvement in the acute stroke care management pathway in the Rhône region, following the advent of EVT and the actions undertaken in the Rhône area in addition to those implemented at the national level.

These results may be compared with those of a national French cross-sectional survey conducted in 2011 and repeated in 2016 i.e. approximately at the same periods [16]. The decrease in delay from hospital admission to imaging observed herein was not found in the national survey (1 h54 [54mn-3 h42] vs. 1 h42 [42mn-3 h48] in 2011 and 2016 respectively), and access to a reperfusion therapy increased from 8.6 to 14.3% at the national level, vs. 9 to 23% in the Rhône County. The absence of improvement in pre-hospital management times was also observed at the national level as in most other studies worldwide [17, 18].

Most experts agree that if ischemic stroke patients were effectively and timely managed, at least one quarter of them would benefit from a reperfusion therapy [19]. The increase in bed capacity and development of EVT are indeed key factors to improve reperfusion therapy, however, complementary organizational actions have to be implemented to facilitate access to these effective techniques. A recent meta-analysis of 86 studies analyzed the efficacy of various interventions for improving acute stroke management, of which 17 focused on pre-hospital, 56 on in-hospital, and 13 on total delays [20]. Significant improvement was associated with new transportation protocols, educational and training programs, and comprehensive pre-hospital stroke code. Pre-notification, and in-hospital organizational programs were also found to significantly reduce in-hospital management times. However, most interventions evaluated in randomized studies concerned only one step of the care pathway.

The main strength of the present study is that it relied on comprehensive data specifically gathered in a research context over a defined catchment area which strongly reduces the risk of selection biases. Another strength is that we had a one-year follow-up to measure the benefit not only on acute care but also on long-term disability. We observed a significant improvement in 1 year post-stroke disability.

One limitation is that this pragmatic design provides an estimate of the overall effectiveness of all actions but not specifically of every action. However, we were able to measure the effectiveness of some of the sets of the actions within randomized or before-after studies. Firstly, the efficacy of our interactive and multifaceted training program targeting emergency professionals was significantly associated with an increased access to thrombolysis, especially within 4h30 by increasing triage nurses and emergency physician awareness on the consequences regarding patients’ long-term disability [12]. This is consistent with the study of Paul et al. that explored barriers to stroke thrombolysis implementation and showed the importance of interactive and competency-based training, and staff performance feedback. Secondly, we also conducted a before-and-after study with a control group which found a statistically significant, although limited in magnitude, effectiveness of the regional campaign [14]. Another limitation is the impossibility of comparing stroke severity measured by the NIHSS between the two periods due to the fact that this score was often not measured by emergency departments’ physicians in 2006-7. However, the national data show that the NIHSS score of ischemic stroke patients at the acute phase stayed stable over time [16]. Furthermore, the improved presence of NIHSS score in the hospital records observed in the second period is an improvement in itself. Regarding 1 year disability, a part of its improvement may also be explained by the implementation of two rehabilitation units in the County between the two survey-periods, one of which being specialized in neurology. The last limitation of this work as in most pragmatic studies in health services research is the importance of the local organization which may impact the reproducibility of interventions and the generalizability of results as it is known that implementation and effectiveness of complex intervention are dependent on their environment [21].

## Conclusion

Between 2006–7 and 2015–16, there was a 3.2 fold increase in access to reperfusion therapy, mainly linked to a decrease of in-hospital management times and a parallel significant improvement in disability at 1 year after stroke. Regarding pre-hospital management, there was a large increase in patients calling ECC although the average delay for ECC calling has not yet decreased. This study provides evidence on the overall effectiveness of combining several sets of actions at different levels of the acute stroke care pathway to improve not only access and time to reperfusion therapy at the acute phase but also long-term disability.

## Data Availability

The datasets used and/or analyzed during the current study are available from the corresponding author on reasonable request.

## Abbreviations

IVT: Intravenous thrombolysis
EVT: Endovascular treatment
ECC: Centralized emergency call center
mRS: Modified Rankin Scale
NIHSS score: National Institute of Health Stroke Scale score
IQR: Interquartile range
SMUR: Service Mobile d’Urgence
